# Anticoagulant Management After Emergency Surgery or Major Bleeding in Anticoagulated Patients—Results of the Prospective RADOA Registry

**DOI:** 10.3390/ph18020170

**Published:** 2025-01-26

**Authors:** Jana Last, Ingvild Birschmann, Simone Lindau, Stavros Konstantinides, Oliver Grottke, Ulrike Nowak-Göttl, Barbara Zydek, Christian von Heymann, Jan Beyer-Westendorf, Sebastian Schellong, Patrick Meybohm, Andreas Greinacher, Eva Herrmann, Edelgard Lindhoff-Last

**Affiliations:** 1 Coagulation Research Centre Bethanien Hospital, 63089 Frankfurt, Germany; b.zydek@ccb.de (B.Z.); e.lindhoff-last@ccb.de (E.L.-L.); 2 Deutsches Herzzentrum der Charité (DHZC) Berlin, Department of Cardiology, Angiology and Intensive Care Medicine, Charité Berlin, 12203 Berlin, Germany; 3 Institute for Laboratory and Transfusion Medicine, Heart and Diabetes Centre, Ruhr University, 44801 Bochum, Germany; ibirschmann@hdz-nrw.de; 4 Department of Anaesthesiology, Intensive Care Medicine and Pain Therapy, University Hospital Frankfurt, 60596 Frankfurt, Germany; simone.lindau@uk-koeln.de; 5 Center for Thrombosis and Hemostasis (CTH), University Medical Center of the Johannes Gutenberg University Mainz, 55122 Mainz, Germany; stavros.konstantinides@unimedizin-mainz.de; 6 Department of Anaesthesiology, RWTH Aachen University Hospital, 52062 Aachen, Germany; ogrottke@ukaachen.de; 7 Institute of Clinical Chemistry, Thrombosis & Hemostasis Treatment Centre, University Hospital, Kiel-Lübeck, 24105 Kiel, Germany; ulrike.nowak-goettl@uksh.de; 8 Coagulation Centre at the Cardiology Angiology Centre Bethanien Hospital (CCB), 63089 Frankfurt, Germany; 9 Department of Anaesthesia, Intensive Care Medicine, Emergency Medicine and Pain Therapy, Vivantes Klinikum im Friedrichshain, 10249 Berlin, Germany; christian.heymann@vivantes.de; 10 Department of Medicine 1, Division of Thrombosis & Hemostasis, Dresden University Clinic, 01307 Dresden, Germany; jan.beyer@uniklinikum-dresden.de; 11 Medical Department 2, Municipal Hospital, 01129 Dresden, Germany; sebastian.schellong@klinikum-dresden.de; 12 Department of Anaesthesiology, Intensive Care, Emergency and Pain Medicine, University Hospital Würzburg, 97080 Würzburg, Germany; meybohm_p@ukw.de; 13 Institute for Transfusions Medicine, Universitätsmedizin Greifswald, 17489 Greifswald, Germany; andreas.greinacher@med.uni-greifswald.de; 14 Institute of Biostatistics and Mathematical Modelling, Goethe University Frankfurt, 60596 Frankfurt, Germany; herrmann@med.uni-frankfurt.de

**Keywords:** emergency surgery, major bleeding, direct oral anticoagulants, vitamin K antagonists, anticoagulation after emergency situation, low-molecular-weight heparin, unfractionated heparin

## Abstract

**Background:** Major bleeding or emergency surgery are the most frequently observed emergency situations in patients anticoagulated with vitamin K antagonists (VKAs) or direct oral anticoagulants (DOACs). The restart of anticoagulation after these situations is a therapeutic dilemma. **Methods:** The prospective RADOA registry is an observational, noninterventional multicenter registry that documents the management of severe bleeding or emergency surgery in patients treated with VKAs or DOACs. In this substudy, we analyzed time point, type, and dosage of anticoagulant resumption after emergency situations. **Results:** Overall, 78 emergency surgery patients and 193 major bleeding patients were analyzed. Median age was similar in the VKA- and DOAC-treated groups (emergency surgery: 77 years, major bleeding: 79 years). Anticoagulants were restarted significantly earlier after emergency surgery compared to major bleeding, with no difference between the VKA and DOAC groups. While patients after cardiothoracic surgery received UFH intravenously, patients with trauma or having received abdominal surgery were mainly treated with prophylactic LMWH s.c.. After major bleeding, the majority of patients were treated with prophylactic LMWH. None of the patients in the emergency surgery group and 17% (4/24) of the major bleeding group with recurrent bleeding (12%, 24/193) experienced recurrent bleeding after restart of anticoagulation. Thromboembolism occurred rarely in both patient groups (emergency surgery: 3%, major bleeding 4%). **Conclusions:** Time points of restart, type, and dosage of anticoagulants are highly diverse in this high-risk patient population. Resumption of prophylactic anticoagulation is associated with a low risk of thrombosis and should be initiated as soon as possible.

## 1. Introduction

For over a decade, anticoagulants have been the most frequently used medication type associated with emergency department (ED) visits for adverse drug events (4.5/1000 population aged ≥ 65 years) [1,2].

The currently approved direct oral anticoagulants (DOACs), which include the factor (F) Xa inhibitors apixaban, rivaroxaban, and edoxaban, as well as the thrombin inhibitor dabigatran, are increasingly used in clinical practice due to two key advantages compared with vitamin K antagonists (VKAs): reduced incidence of major bleeding and simplified perioperative management [3,4].

However, with increased use of DOACs, they remain important contributors to medications associated with ED visits for medication harms [1,5]. About 4–5% of anticoagulated patients present with major bleeding [3] and about 0.5–1% of anticoagulated patients need emergency surgery each year [6]. Fast and adequate supportive care as well as discontinuation of the oral anticoagulant are essential for the management of emergency surgeries and bleeding complications. Moreover, additional measures, such as the administration of procoagulants and/or specific antidotes, may be needed to manage emergency surgery and to control bleeding [7].

The restart of anticoagulation after an emergency situation is a therapeutic dilemma in this high-risk patient population. Not only the timing of restart but also the dosing and type of anticoagulant treatment are therapeutic challenges. The bleeding risk of the restarted anticoagulation needs to be balanced against the thromboembolic risk without anticoagulation. So far, this problem has barely been addressed in the current literature.

We analyzed the anticoagulant management after emergency surgery or major bleeding in anticoagulated patients using data from the prospective German RADOA registry (reversal agent use in patients treated with direct oral anticoagulants or vitamin K antagonists).

## 2. Results

### 2.1. Demographic Data of Included Patients

Overall, 78 anticoagulated patients who received emergency surgery (DOACs: 44, VKAs: 34) and 193 patients with major bleeding (DOACs: 96, VKA: 97) were analyzed. Demographic data of patients are given in Table 1 and Table 2.

In the emergency surgery group, 52% (23/44) of the DOAC-patients were treated with apixaban, 32% (14/44) with rivaroxaban, 7% (3/44) with edoxaban, and 9% (4/44) with dabigatran. In the major bleeding group, 43% (41/96) of the DOAC patients received apixaban, 48% (46/96) rivaroxaban, 5% (5/96) edoxaban, and 4% (4/96) dabigatran. The frequency of DOAC treatments between the bleeding group and the emergency surgery group did not differ significantly (*p* = 0.252) and corresponded to the relative DOAC distribution in Germany at the time the registry had taken place [8]. One patient who was treated with a VKA and apixaban at the same time (medication error) was excluded from this analysis.

Median age was similar in both groups (emergency surgery: 77 years, major bleeding: 79 years) (see Table 1 and Table 2). A significant sex difference between the DOAC- and VKA-treated patients was observed in the emergency surgery group (female DOAC patients: 52% (23/44) vs. female VKA patients: 23% (8/34); *p* = 0.012, see Table 1), while no significant difference was seen in the major bleeding-group (female DOAC patients: 47% (45/96) vs. female VKA patients: 33% (33/97); *p* = 0.079), see Table 2). On admission, acute renal failure occurred to a similar extent in both groups (emergency surgery: DOAC: 5% (2/44) vs. VKA: 9% (3/34), *p* > 0.20; major bleeding: DOAC: 12% (11/96) vs. VKA 7% (7/97), *p* > 0.20, see Table 1 and Table 2).

In the emergency surgery-group, indications for emergency surgery were mainly bone fractures (35%, 27/78) or an acute abdomen (30%, 23/78). Significantly more DOAC-treated patients suffered from bone fractures compared to VKA-treated patients (DOAC: 46%, 20/44 vs. VKA: 21%,7/34; *p* = 0.031). Numerically more VKA-treated patients needed open heart surgery compared to the DOAC-treated patients (VKA: 21%, 7/34 vs. DOAC: 9%, 4/44; *p* = 0.195) (see Table 1).

In the bleeding group, intracranial hemorrhage was more frequently observed in patients treated with VKAs compared to patients receiving DOAC therapy (66% (64/97) vs. 47% (45/96); *p* = 0.009). In contrast, gastrointestinal bleeding was found less frequently in patients treated with VKAs compared to patients treated with DOACs (20% (19/97) vs. 32% (31/96); *p* = 0.050) (see Table 2).

Atrial fibrillation was the predominant indication for oral anticoagulation in both groups: emergency surgery 74% (58/78), major bleeding 78% (150/193). The median CHADS-VASC score was 5 in both groups. The median bleeding risk score according to the modified HASBLED score (HASBLED score excluding the criterion “stability of INR”) was 2 in the emergency surgery group and 3 in the major bleeding group. Concomitant treatment with antiplatelet drugs or anti-inflammatory drugs was similarly distributed in both groups (antiplatelet drugs: emergency surgery 14% (11/78) vs. major bleeding 17% (33/193); anti-inflammatory drugs: emergency surgery 5% (4/78) vs. major bleeding 7%, (14/193) (see Table 1 and Table 2).

### 2.2. Start and Type of Anticoagulants Used After the Emergency Situation

**Figure 1:**
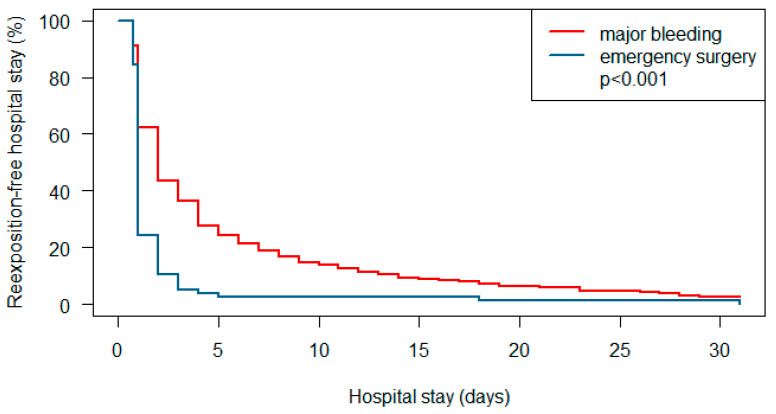
Rates of reexposure-free hospital stay in patients with major bleeding compared to patients with emergency surgery.

After analyzing a combined endpoint of anticoagulation restart, hospital discharge, or death, 97.4% of patients in the emergency surgery group had already reached this endpoint on day 5 after hospital admission compared to 76% of the patients with major bleeding (log-rank test for the whole time range *p* < 0.001, see Figure 1).

**Figure 2:**
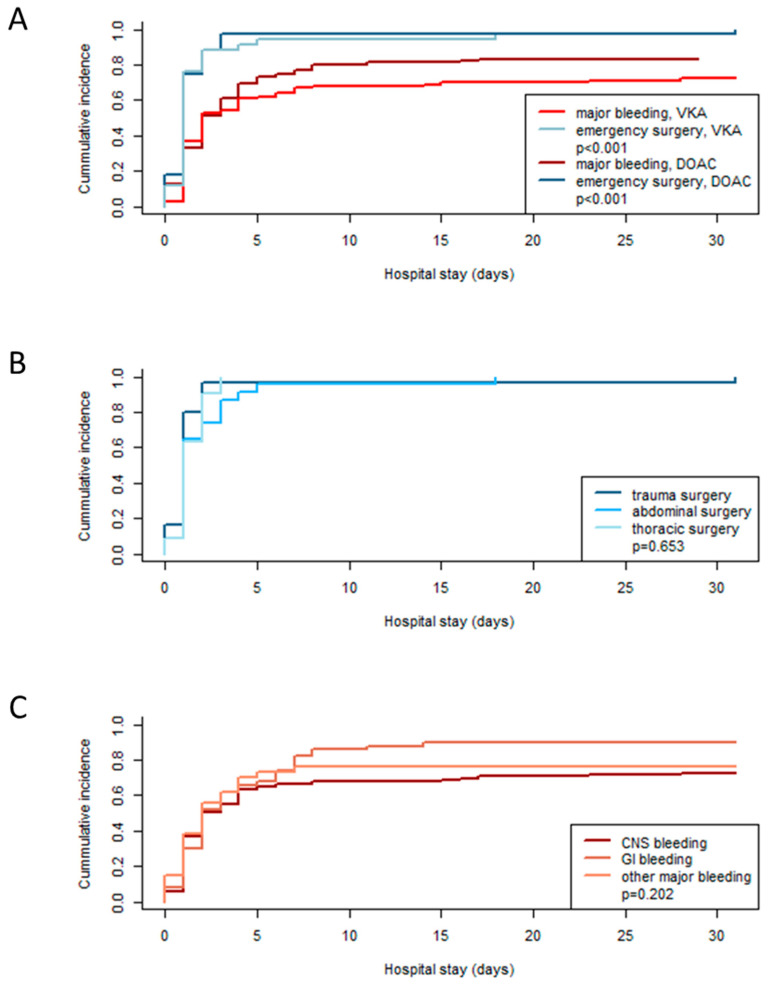
Timing of the restart of anticoagulation. (**A**) Comparisons of the timing of the restart of anticoagulation in DOAC-treated patients and VKA-treated patients with major bleeding or emergency surgery. DOAC = direct oral anticoagulant, VKA = vitamin K antagonist. (**B**) Restart of anticoagulation in patients with emergency surgery depending on the type of emergency surgery. (**C**) Restart of anticoagulation in patients with major bleeding depending on the location of major bleeding. CNS = central nervous system, GI = gastrointestinal.

When comparing the VKA and DOAC groups, anticoagulant treatment was started significantly earlier after emergency surgery compared to major bleeding, with no difference between the respective VKA and DOAC subgroups (see Figure 2A, *p* < 0.001). In the emergency surgery group, the first reexposure to an anticoagulant was observed in 88% of the patients at day 2 and in 96% at day 5 after hospital admission compared to 52% at day 2 and 67% at day 5 in the major bleeding group.

Restart of anticoagulation did not differ in patients after trauma, abdominal, or thoracic surgery (see Figure 2B, *p* = 0.653). Moreover, anticoagulation resumption was not different in patients after CNS bleeding compared to the other types of major bleeding (see Figure 2C, *p* = 0.202).

Patients were reexposed with different types of anticoagulants.

**Figure 3:**
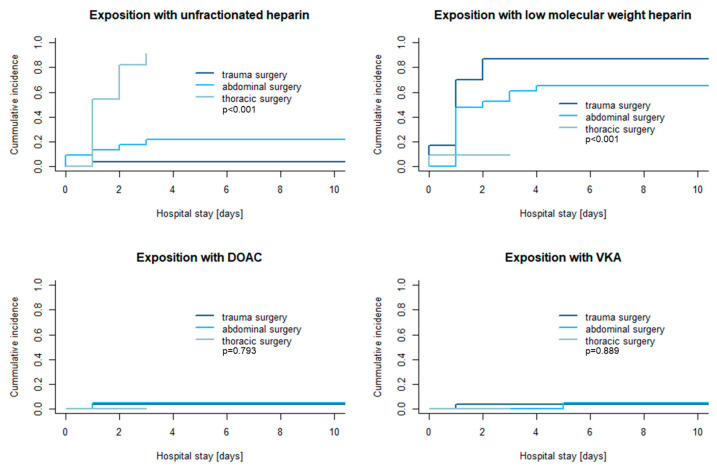
First exposure to different types of anticoagulants in patients with major surgery within 10 days after hospital admission. The heterogeneous group of patients with other surgery types was excluded from this evaluation. DOAC = direct oral anticoagulant, VKA = vitamin K antagonist.

After emergency surgery, significant differences were observed for exposure to unfractionated heparin (UFH) or low-molecular-weight heparin (LMWH) until day 10 after hospital admission. While patients after thoracic surgery (predominantly open heart surgery) received UFH intravenously, patients with trauma or abdominal surgery were mainly treated with LMWH s.c. (*p* < 0.001, see Figure 3). In the majority of the emergency surgery patients, prophylactic LMWH was started (41% (32/78)), while in 35% (27/78) of patients, LMWH was initiated at higher dosages. When intravenous UFH was initiated, 32% (25/78) of the patients received dosages < 600IU/h and 14% (11/78) dosages > 600 IE/h.

Exposure to DOACs or VKAs was rarely observed after emergency surgery (see Figure 3 and Table 3).

**Figure 4:**
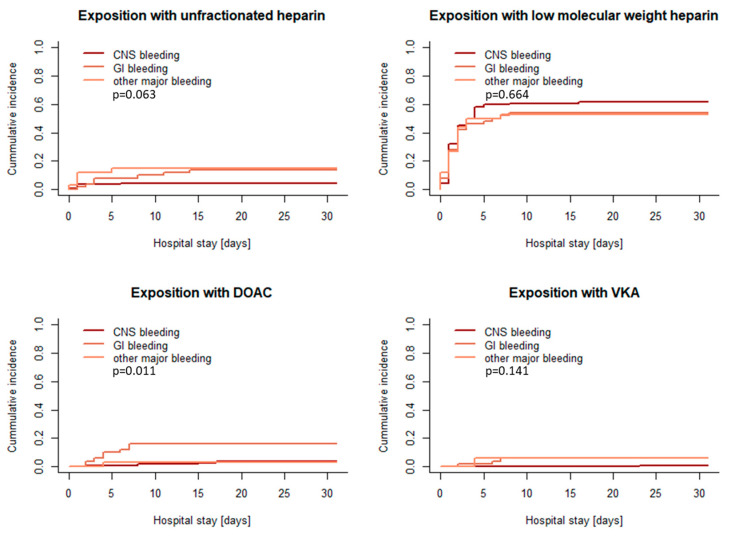
First exposure to different types of anticoagulants in patients with major bleeding within 30 days after hospital admission. CNS = central nervous system, GI = gastrointestinal, DOAC = direct oral anticoagulant, VKA = vitamin K antagonist.

After major bleeding, the majority of patients were exposed to LMWH (see Figure 4 and Table 4). While 46% of the patients (89/193) were started with prophylactic doses of LMWH during the first 10 days, 14% of the patients (27/193) received higher LMWH doses when anticoagulation was initiated. Anticoagulation was rarely restarted with intravenous UFH. In 7% of patients with major bleeding (14/193), UFH dosages < 600 IU/h were used, while in 3% of patients (5/193), anticoagulation was initiated with UFH dosages > 600 IU/h.

Although DOAC exposure was rarely used after severe bleeding, treatment with a DOAC was observed significantly more often in the GI bleeding group compared to the other bleeding types (see Figure 4 and Table 4).

In addition, two patients were exposed to fondaparinux: one patient with CNS bleeding started treatment on day 1 and a second patient with CNS bleeding after day 10.

### 2.3. Occurrence of Bleeding After Acute Emergency Situations

One of the 78 patients in the emergency surgery group (1%) experienced postoperative bleeding without being reexposed to an anticoagulant.

Recurrent bleeding was documented in 24/193 (12%) patients with initial major bleeding. In sum, 14 patients had been treated with DOACs and 10 with VKAs at baseline. Recurrent bleeding in four patients (4/24, 17%) was categorized as being related to an anticoagulant reexposure. In two patients, multiple antithrombotic treatments had been applied at the time of recurrent bleeding (patients 1 and 2), while recurrent intracranial hemorrhage occurred in two other patients while they were treated with the LMWH enoxaparin (patients 3 and 4). In an additional statistical analysis, no significant predictors of recurrent bleeding upon admission, such as age, body mass index (BMI), type of bleeding, cancer, renal impairment, or vascular risk factors, could be identified. For more details, see Table 5.

### 2.4. Occurrence of Thrombosis After Acute Emergency Situations

Two patients developed a thromboembolic event after emergency surgery (2/78, 3%). One patient presented with a deep vein thrombosis despite application of enoxaparin 40 mg once daily postoperatively. The second patient developed recurrent arterial occlusion after peripheral bypass surgery despite anticoagulation with UFH intravenously.

Thromboses occurred to a similar extent in 7 of 193 patients (4%) after major bleeding. Four of seven patients (57%) suffered from venous thromboembolism without reexposure to an anticoagulant: One patient presented with superficial vein thrombosis on day 8 after admission and three patients developed a pulmonary embolism.

Three of seven patients (43%) developed a thromboembolic complication despite restart of anticoagulation. Two patients in the CNS bleeding group developed deep vein thrombosis despite prophylactic treatment with enoxaparin 40 mg once daily. One patient presented with an ischemic stroke caused by endocarditis on day 14 after admission despite intravenous UFH treatment.

## 3. Discussion

To our knowledge, the RADOA registry is the first multicenter registry to prospectively analyze the reinitiation of anticoagulation in either DOAC- or VKA-anticoagulated patients after emergency situations, including major bleeding or emergency surgery within 24 h. As expected, the included patients represented a rather elderly population, with a median age between 77 and 79 years. Similar age distributions were observed in other registries and meta-analyses focusing on anticoagulated patients in these emergency situations [9,10,11].

The most frequent indication for emergency surgery was bone fractures, followed by acute abdomen. These results are in accordance with the French observational GIHP-NACO registry, which analyzed the management of urgent invasive procedures in DOAC-treated patients [11].

In the bleeding group, intracranial hemorrhage was most frequently observed in the VKA and the DOAC groups, while gastrointestinal bleeding was the second-most common type of bleeding. Both a review and a Dutch registry showed a similar distribution of major bleeding in DOAC-treated patients [10,12].

Restarting anticoagulation after emergency situations is an increasing therapeutic challenge in clinical routine. So far, there is a lack of scientific evaluations in prospective observational studies addressing the time point, type, and dose of the anticoagulation resumption.

In our registry, anticoagulant treatment was started significantly earlier after emergency surgery compared to major bleeding, with no difference between the VKA and DOAC groups. Restart of anticoagulation did not differ in patients after trauma, abdominal, or cardiothoracic surgery. Moreover, anticoagulation resumption was not different in patients after CNS bleeding compared to the other types of major bleeding. By day 2 after admission, anticoagulation had already been restarted in 89% of patients with emergency surgery, in contrast to 52% of patients with initial major bleeding. This trend continued until day 10 after admission: while 96% (4/78) of the emergency surgery patients had been reexposed, only 74% (143/193) of the major bleeding patients had received an anticoagulant.

Subcutaneously applied LMWHs were the preferred anticoagulants when anticoagulation was restarted. In cases of a high bleeding risk, such as in emergency patients after cardiothoracic surgery, UFH was applied intravenously due to its shorter half-life. Anticoagulant dosages were highly diverse and depended on the individual clinical situations of the patients. At the time of restarting anticoagulation, prophylactic doses of LMWH and UFH were preferred. Recurrent bleeding risks due to restart of anticoagulation and thromboembolic risks after restart of anticoagulation were low in both patient groups: in the emergency surgery group, no recurrent bleeding occurred after the restart of anticoagulation, in the major bleeding group, only 17% (4/24) of patients experienced recurrent bleeding after the restart of anticoagulation, while 83% (20/24) developed new bleeding episodes before the resumption of anticoagulation. Interestingly, thromboembolic complications were rarely observed in this fragile, old patient population (emergency surgery group: 3%, major bleeding group: 4%) and some of the thromboembolic events occurred despite the restart of anticoagulation [9].

These data are in accordance with findings of a meta-analysis focusing on reversal agent use in DOAC-anticoagulated patients with major bleeding, which observed a relatively low thromboembolic rate in patients treated with four-factor prothrombin complex concentrates (PCCs) (4.3%) and idarucizumab (3.8%).

The RADOA registry is subject to all limitations that are typical for observational studies, including patient selection bias, subjective and non-standardized treatment decisions, and reporting bias. Moreover, the small number of included patients limits the transferability of the results to the corresponding patient population [13]. Based on the registry data collected so far, it is unfortunately not possible to conclude whether the treatment approaches chosen by the treating physicians in the registry have been the best medical treatment. Due to the emergency situations and the heterogeneous patient population, it is questionable whether a randomized, controlled study on the restart of anticoagulation is feasible and useful.

Despite these problems, our registry is the only one that has evaluated the start, type, and intensity of anticoagulation after different emergency situations in detail. Most of the current management guidelines do not give evidence-based recommendations on anticoagulation after these emergency situations, possibly because there have been hardly any evaluations of these difficult management situations in other registries and studies to date.

In the recently published WSES guideline on the management of trauma, it is recommended that venous thromboembolism prophylaxis with LMWH or UFH be administered as soon as possible in the trauma setting according to renal function, weight of the patient, and bleeding risk, which is in agreement with our findings in the emergency surgery group [14]. After traumatic brain injury, the decision about whether and when to resume antithrombotic therapies after the acute phase needs a case-by-case evaluation [15].

A previously published review dealing with these emergency situations in anticoagulated patients recommends what was confirmed by our evaluations: Levy et al. reviewed the management of major bleeding and emergency surgery in DOAC-treated patients [16]. They recommend that after a major anticoagulant-related bleed, clinicians should consider resuming anticoagulant therapy, because the evidence consistently shows that this strategy confers a reduction in stroke and overall mortality that offsets any increase in recurrent bleeding. They suggest that the timing of anticoagulant resumption should be individualized on the basis of the potential for bleeding recurrence.

In contrast to prophylactic anticoagulation with s.c. LMWH and/or i.v. UFH, which is frequently restarted after acute events, reintroduction of therapeutic oral anticoagulant therapy after major bleeding is often complicated and inconsistently approached [10]. This agrees with the RADOA registry findings, which showed that DOAC or VKA reintroduction were infrequently observed before hospital discharge. Therefore, a randomized study that investigates the optimal time point at which to switch from parental prophylactic to oral therapeutic anticoagulation at 1, 2, or 4 weeks after admission is urgently needed.

## 4. Materials and Methods

### 4.1. Study Design and Oversight

The prospective RADOA registry is a German observational, noninterventional, open-label, investigator-initiated, multicenter registry that documents the management of severe bleeding and/or emergency surgery in patients treated with the vitamin K antagonist phenprocoumon or DOACs.

The issues and purposes of the registry have been described previously [13,17,18,19,20,21]. Patients were followed prospectively up to 30 days after admission to hospital. Participating centers were included only if they had 24 h interdisciplinary teams available to manage anticoagulant-related bleeding or emergency surgery in anticoagulated patients in specialized units (i.e., emergency departments and intensive care units). Patients were recruited consecutively at 10 German centers from April 2014 to March 2018.

This manuscript focuses on anticoagulant management after acute emergency events. Timing of restart of anticoagulation, types of anticoagulants used for anticoagulation restart, and their safety and efficacy were analyzed up to 30 days after admission to hospital or until discharge or death.

### 4.2. Patients

The inclusion criteria of the RADOA registry were:

- Age > 18 years.
- Patients anticoagulated with DOACs or phenprocoumon with major bleeding or needing an urgent surgical intervention within 24 h after admission.

For more details, see the previously published literature [13,18].

### 4.3. Ethics

Approval of the study protocol was required by all relevant institutional review boards. External independent monitoring ensured 100% site source data validity.

Due to the emergency nature of the conditions under investigation, patient information and informed consent should not interfere with or delay acute treatment. With the approval of all ethics committees and institutional review boards, written informed consent was obtained from patients after the acute management phase. In the event of patient inability to provide written informed consent, this was obtained from his/her legal representative. Data of patients who remained unconscious or died before a legal representative had been appointed were also included. This was explicitly approved by the ethics boards to prevent major bias caused by exclusion of the most severely affected patients [13,18]. The study complied with the Declaration of Helsinki.

ClinicalTrials.gov identifier: NCT01722786

### 4.4. Statistics

Categorical data are reported as absolute and relative frequencies, and continuous data and scores are reported as medians with quartiles to indicate the interquartile range.

Combined time-to-event endpoint was evaluated with log-rank tests and Kaplan–Meier curves. Competing risk analysis was performed using Gray’s test, and cumulative incidence curves are shown for the endpoints of interest (reexposure to anticoagulants, using hospital discharge and in-hospital death as competing events). Predictors of recurrent bleeding were analyzed by univariate logistic regression.

All statistical tests were explorative. A two-sided approach and a significance level of alpha = 5% were used.

Statistical analysis was conducted using R (R Software Version 4.4 for Statistical Computing, Vienna, Austria) using the survival and cmprsk packages.

## 5. Conclusions

Care of anticoagulated patients in the acute setting is inconsistent, reflecting the diversity of presentation. As the prevalence of DOAC use will further increase with the aging of the worldwide population, further study and targeted educational efforts are needed to drive more evidence-based care of these patients.

## Figures and Tables

**Table 1:** Characteristics of emergency surgery patients at baseline. Results are given in percentages or as medians with interquartile ranges.

	Total (n = 78)	DOAC ^4^ (n = 44)	VKA (n = 34)	*p*-Values ^1^
**Demographic data**				
Female sex	31 (40%)	23 (52%)	8 (23%)	0.012
Age	77 (68–83)	76 (69–83)	77 (67–82)	>0.20
Acute renal failure	5 (6%)	2 (5%)	3 (9%)	>0.20
**Surgery type**				
Traumatic	31 (40%)	24 (55%)	7 (21%)	0.003
Fractures ^5^	27 (35%)	20 (46%)	7 (21%)	0.031
Acute abdomen	23 (30%)	12 (27%)	11 (32%)	>0.20
GI tract	18 (23%)	9 (21%)	9 (27%)	>0.20
CNS	3 (4%)	2 (5%)	1 (3%)	>0.20
Abscess	1 (1%)	1 (2%)	0 (0%)	--
Open-heart surgery	11 (14%)	4 (9%)	7 (21%)	0.195
Vascular	7 (9%)	2 (5%)	5 (15%)	>0.20
Other	6 (8%)	3 (7%)	3 (9%)	>0.20
**Co-medication, indication for anticoagulation and bleeding risk**				
Antiplatelet drugs	11 (14%)	6 (14%)	5 (15%)	>0.20
Anti-inflammatory drugs	4 (5%)	1 (2%)	3 (9%)	>0.20
**Indication**				0.155
Atrial fibrillation	58 (74%)	34 (77%)	24 (71%)	
Venous thrombosis	5 (6%)	4 (9%)	1 (3%)	
Thrombosis prophylaxis	4 (5%)	1 (2%)	3 (9%)	
Arterial thrombosis	4 (5%)	1 (2%)	3 (9%)	
Artificial heart valve	2 (3%)	0 (0%)	2 (6%)	
Other	5 (6%)	4 (9%)	1 (3%)	
CHADS-VASC Score	5.0 (4.0–6.0)	5.0 (4.0–6.0)	5.0 (3.8–6.3)	>0.20
HASBLED Score ^2^	3.0 (2.0–3.0)	2.0 (2.0–3.0)	3.0 (3.0–3.0)	0.126
HASBLED Score modified ^3^	2.0 (2.0–3.0)	2.0 (3.0–4.0)	3.0 (3.0–3.0)	>0.20
**Previous history**				
**Cancer**				>0.20
None	63 (81%)	36 (82%)	27 (79%)	
Previous	8 (10%)	4 (9%)	4 (12%)	
Acute	7 (9%)	4 (9%)	3 (9%)	
Previous Stroke	14 (18%)	6 (14%)	8 (24%)	>0.20
Previous myocardial infarction	9 (12%)	4 (9%)	5 (15%)	>0.20
Previous coronary disease	11 (14%)	6 (14%)	8 (24%)	>0.20
**Previous renal impairment**				0.04
No	55 (77%)	34 (85%)	21 (68%)	
GFR > 90 mL/min/1.73 m^2^	2 (3%)	2 (5%)	0	
GFR 60–89 mL/min/1.73 m^2^	2 (3%)	1 (3%)	1 (3%)	
GFR 30–59 mL/min/1.73 m^2^	8 (11%)	3 (8%)	5 (16%)	
GFR 15–29 mL/min/1.73 m^2^	4 (6%)	0	4 (13%)	
GFR < 15 mL/min/1.73 m^2^	0	0	0	
**Respiratory function on admission**				>0.20
Unknown or normal	68 (87%)	39 (89%)	29 (85%)	
Dyspnea	6 (8%)	2 (5%)	4 (12%)	
Apnea	4 (5%)	3 (7%)	1 (3%)	

^1^ Explorative *p*-values: two-sided tests (Fisher test, Wilcoxon-Mann-Whitney test) for comparison of the DOAC- vs. VKA-group without significance correction for multiple testing. ^2^ Modified HASBLED score (i.e., HASBLED score excluding the criterion “stability of INR”) was used in patients treated with DOAC while the HASBLED score including stability of INR was used in patients treated with VKA. ^3^ Modified HASBLED score (i.e., HASBLED score excluding the criterion “stability of INR”) was used in all patients. ^4^ Dosing data were available for 42 of 44 patients treated with DOACs on admission. 38% of these patients received a reduced DOAC-dose (16/42). ^5^ Subgroup of traumatic surgery. Abbreviations: DOAC = direct oral anticoagulant, VKA = vitamin K antagonist, INR = international normalised ratio.

**Table 2:** Characteristics of bleeding patients at baseline. Results are given in percentages or as medians with interquartile ranges.

	Total (n = 193)	DOAC ^4^ (n = 96)	VKA (n = 97)	*p*-Values ^1^
**Demographic data**				
Female sex	115 (60%)	45 (47%)	33 (33%)	0.079
Age	79 (73–84)	81 (74–84)	78 (73–84)	>0.20
Acute renal failure	18 (9%)	11 (12%)	7 (7%)	>0.20
**Bleeding type**				
Intracranial/intraspinal	109 (57%)	45 (47%)	64 (66%)	0.009
Gastrointestinal tract	50 (26%)	31 (32%)	19 (20%)	0.050
Intramuscular	4 (2%)	2 (2%)	2 (2%)	>0.20
Retroperitoneal	4 (2%)	2 (2%)	2 (2%)	>0.20
Intra-articular	2 (1%)	2 (2,1%)	0 (0%)	--
Intraocular	1 (0.5%)	0 (0%)	0 (0.0%)	--
Other	23 (12%)	14 (15%)	9 (9%)	>0.20
Unknown	1 (0.5%)	1 (1%)	0 (0%)	--
**Co-medication, indication for anticoagulation and bleeding risk**				
Antiplatelet drugs	33 (17%)	17 (18%)	16 (17%)	>0.20
Anti-inflammatory drugs	14 (7%)	6 (6%)	8 (8%)	>0.20
**Indication**				0.155
Atrial fibrillation	150 (78%)	77 (80%)	73 (75%)	
Venous thrombosis	14 (7%)	6 (6%)	8 (8%)	
Thrombosis prophylaxis	4 (2%)	2 (2%)	2 (2%)	
Arterial thrombosis	3 (2%)	0 (0%)	3 (3%)	
Artificial heart valve	8 (4%)	1 (1%)	7 (7%)	
Other	9 (5%)	7 (7%)	2 (2%)	
CHADS-VASC Score	5.0 (4.0–6.0)	5.0 (4.0–6.0)	5.0 (4.0–6.0)	>0.20
HASBLED Score ^2^	3.0 (2.0–4.0)	3.0 (2.0–3.0)	3.0 (2.0–4.0)	>0.20
HASBLED Score modified ^3^	3.0 (0.0-6.0)	3.0 (1.0-6.0)	3.0 (0.0-6.0)	>0.20
**Previous history**				
**Cancer**				0.11
None	145 (75%)	77 (80%)	68 (70%)	
Previous	40 (21%)	14 (15%)	4 (12%)	
Acute	8 (4%)	5 (5%)	3 (3%)	
Previous Stroke	31 (16%)	15 (16%)	16 (16%)	>0.20
Previous myocardial infarction	20 (10%)	11 (11%)	9 (9%)	>0.20
Previous coronary disease	28 (15%)	17 (18%)	11 (11%)	>0.20
**Previous renal impairment**				>0.20
No	138 (75%)	71 (77%)	67 (73%)	
GFR >90 mL/min/1.73 m^2^	5 (3%)	2 (2%)	3 (3%)	
GFR 60–89 mL/min/1.73 m^2^	5 (3%)	2 (2%)	3 (3%)	
GFR 30–59 mL/min/1.73 m^2^	23 (13%)	11 (12%)	12 (13%)	
GFR 15–29 mL/min/1.73 m^2^	8 (4%)	4 (4%)	4 (4%)	
GFR <15 mL/min/1.73 m^2^	5 (3%)	2 (2%)	3 (3%)	
**Respiratory function on admission**				>0.20
Unknown or normal	156 (81%)	81 (84%)	75 (77%)	
Dyspnea	29 (15%)	13 (14%)	16 (16%)	
Apnea	8 (4%)	2 (2%)	6 (6%)	

^1^ Explorative *p*-values: two-sided tests (Fisher test, Wilcoxon-Mann-Whitney test) for comparison of the DOAC- vs. VKA-group without significance correction for multiple testing. ^2^ Modified HASBLED score (i.e., HASBLED score excluding the criterion “stability of INR”) was used in patients treated with DOAC while the HASBLED score including stability of INR was used in patients treated with VKA. ^3^ Modified HASBLED score (i.e., HASBLED score excluding the criterion “stability of INR”) was used in all patients. ^4^ Dosing data were available for 88 of 96 patients treated with DOACs on admission. 50% of these patients received a reduced DOAC-dose (44/88). Abbreviations: DOAC = direct oral anticoagulant, VKA = vitamin K antagonist, INR = international normalized ratio.

**Table 3:** Emergency surgery group: reexposure to anticoagulant treatment during the first 10 days of hospital stay (multiple treatments are possible).

	Trauma *n* = 30	Abdominal *n* = 23	Thoracic *n* = 11	Other *n* = 14	*p*-Value
No reexposure	1 (3%)	1 (4%)	0 (0%)	1 (7%)	>0.200
Unfractionated heparin	8 (27%)	9 (39%)	10 (91%)	11 (79%)	<0.001
treatment start in ICU	7	8	10	7	
treatment start in normal ward	1	1	0	4	
Low-molecular-weight heparin	26 (87%)	19 (83%)	4 (36%)	10 (71%)	0.011
treatment start in ICU	10	11	1	2	
treatment start in normal ward	16	8	3	8	
DOAC	2 (7%)	2 (9%)	0 (0%)	0 (0%)	>0.200
treatment start in ICU	0	0	0	0	
treatment start in normal ward	2	2	0	0	
VKA	1 (3%)	2 (9%)	4 (36%)	2 (14%)	0.118
treatment start in ICU	1	0	1	1	
treatment start in normal ward	0	2	3	1	

GI = gastrointestinal, ICU = intensive care unit, DOAC = direct oral anticoagulant, VKA = vitamin K antagonist.

**Table 4:** Major bleeding-group: reexposure to anticoagulant treatment during the first 10 days of hospital stay (multiple treatments are possible).

	Cerebral *n* = 109	GI *n* = 50	Other *n* = 34	*p*-Value
No reexposure	35 (32%)	7 (14%)	8 (24%)	0.108
Unfractionated heparin	10 (9%)	6 (12%)	6 (18%)	>0.200
treatment start in ICU	9	4	6	
treatment start in normal ward	1	2	0	
Low molecular weight heparin	67 (62%)	29 (58%)	21 (62%)	>0.200
treatment start in ICU	48	6	9	
treatment start in normal ward	19	24	13	
DOAC	2 (2%)	17 (34%)	2 (6%)	<0.001
treatment start in ICU	1	2	0	
treatment start in normal ward	1	15	2	
VKA	0 (0%)	5 (10%)	2 (6%)	0.002
treatment start in ICU	0	0	0	
treatment start in normal ward	0	5	2	

GI = gastrointestinal, ICU = intensive care unit, DOAC = direct oral anticoagulant, VKA = vitamin K antagonist.

**Table 5:** Recurrent bleeding after restart of antithrombotic therapy (anticoagulants and/or aggregation inhibitors) in patients with major bleeding.

	Age (Years), Gender	Bleeding Group	Initial OAC at Baseline	Start of a Platelet Inhibitor (Days After Admission)	Restart of Anticoagulation (Days After Admission)	Antithrombotic Therapy at the Time of Recurrent Bleeding	Occurrence and Type of Recurrent Bleeding (Days after Admission)
Pat. 1	84, m	other	rivaroxaban + ASS	ASS 100 mg ≥ day 5	UFH 100–1100 U/h i.v. day 1–4 enoxaparin 2 × 40 mg ≥ day 4	enoxaparin 2 × 40 mg + ASS 100 mg	day 5 epistaxis
Pat. 2	71, f	GI	rivaroxaban + clopidogrel + ASS	clopidogrel 75 mg ≥ day 1 ASS 100 mg ≥ day 3	rivaroxaban 1 × 15 mg ≥ day 5	clopidogrel 75 mg + ASS 100 mg + rivaroxaban 1 × 15 mg	day 5 GI
Pat. 3	89, f	ICH	phenprocoumon		enoxaparin (1 × 20)–(1 × 40) mg s.c. alternating with UFH 400–600 U/h i.v. ≥ day 1	enoxaparin ^1^	day 7 ICH
Pat. 4	66, m	ICH	rivaroxaban		enoxaparin 1 × 40 mg ≥ day 1	enoxaparin 1 × 40 mg	day 4, 6, 7 ^2^ ICH

m = male, f = female, GI = gastrointestinal, ICH = intracranial hemorrhage, OAC = oral anticoagulant, UFH = unfractionated heparin, ASS = acetylsalicylic acid, i.v. intravenously. ^1^ Dosage unknown, ^2^ died on day 13 after admission.

## Data Availability

Data are contained within the article.
